# Systemic antibiotic sales and WHO recommendations, India

**DOI:** 10.2471/BLT.22.287908

**Published:** 2022-08-22

**Authors:** Aashna Mehta, Petra Brhlikova, Patricia McGettigan, Allyson M Pollock, Peter Roderick, Habib Hasan Farooqui

**Affiliations:** a Public Health Foundation of India, Gurugram, India.; b Population Health Sciences Institute, Newcastle University, Baddiley-Clark Building, Newcastle upon Tyne, NE2 4AX, England.; c William Harvey Research Institute, Queen Mary University of London, London, England.; d College of Medicine, Qatar University, Doha, Qatar.

## Abstract

**Objective:**

To analyse sales of fixed-dose combination and single antibiotics in India in relation to World Health Organization (WHO) recommendations and national regulatory efforts to control antibiotic sales.

**Methods:**

We extracted data on sales volumes of systemic antibiotics in India from a market research company sales database. We compared the market share of antibiotic sales in 2020 by WHO AWaRe (Access, Watch and Reserve) category and for those under additional national regulatory controls. We also analysed sales of fixed-dose combinations that were: formally approved for marketing or had a no-objection certificate; on the national essential medicines list; and on the WHO list of not-recommended antibiotics.

**Findings:**

There were 78 single and 112 fixed-dose combination antibiotics marketed in India, accounting for 7.6 and 4.5 billion standard units of total sales, respectively. Access, Watch and Reserve antibiotics comprised 5.8, 5.6 and 0.1 billion standard units of total market sales, respectively. All additionally controlled antibiotics were Watch and Reserve antibiotics (23.6%; 2.9 billion standard units of total sales). Fixed-dose combinations on the WHO not-recommended list were marketed in 229 formulations, with 114 formulations (49.8%) having no record of formal approval or no-objection certificate. While there were no not-recommended fixed-dose combinations on the national list of essential medicines, 13 of the top-20 selling antibiotic fixed-dose combinations were WHO not-recommended.

**Conclusion:**

The sale of Watch group drugs, and antibiotics banned or not approved, needs active investigation and enforcement in India. The evidence base underpinning formal approvals and no-objection certificates for not-recommended fixed-dose combinations should be audited.

## Résumé

**Objectif:**

Analyser les ventes d'antibiotiques en association fixe et à dose unique en Inde, à la lumière des recommandations de l'Organisation mondiale de la Santé (OMS) et des efforts de réglementation à l'échelle nationale visant à contrôler la vente d'antibiotiques.

**Méthodes:**

Nous avons prélevé des données relatives aux volumes de vente d'antibiotiques systémiques en Inde dans la base de données commerciales d'une société d'études de marché. Nous avons ensuite comparé la part de marché qu'occupe, en 2020, la vente d'antibiotiques appartenant à chaque catégorie AWaRe (Access, Watch and Reserve) établie par l'OMS, mais aussi de ceux ciblés par des mesures nationales de contrôle supplémentaires. Enfin, nous avons examiné les ventes d'associations fixes: dont la commercialisation a été officiellement approuvée ou qui ont fait l'objet d'un certificat de non-objection; figurant sur la liste nationale de médicaments essentiels; et reprises dans la liste des antibiotiques non recommandés par l'OMS.

**Résultats:**

Nous avons comptabilisé 78 antibiotiques à dose unique et 112 antibiotiques en association fixe sur le marché indien, représentant respectivement 7,6 et 4,5 milliards d'unités standard sur l'ensemble des ventes. Les antibiotiques des catégories Access (dont l'accessibilité est essentielle), Watch (à utiliser sélectivement) et Reserve (de réserve, non recommandés) équivalaient à 5,8 milliards, 5,6 milliards et 0,1 milliard d'unités standard sur le total des transactions. Tous les antibiotiques soumis à des contrôles supplémentaires faisaient partie des catégories Watch et Reserve (23,6%; 2,9 milliards d'unités standard sur l'ensemble des ventes). Des associations fixes déconseillées par l'OMS étaient commercialisées dans 229 formulations, dont 114 (49,8%) ne possédaient ni attestation d'approbation officielle, ni certificat de non-objection. Bien qu'aucune association fixe déconseillée ne soit mentionnée sur la liste nationale de médicaments essentiels, 13 des 20 antibiotiques en association fixe les plus vendus n'étaient pas recommandés par l'OMS.

**Conclusion:**

Il est nécessaire de mener une enquête approfondie en Inde sur la vente de médicaments appartenant à la catégorie Watch et d'antibiotiques interdits ou non approuvés, et de prendre des mesures concrètes. Les preuves sur lesquelles reposent les autorisations officielles et les certificats de non-objection pour les associations fixes non recommandées doivent être vérifiées.

## Resumen

**Objetivo:**

Analizar las ventas de los antibióticos combinados en dosis fijas y en dosis únicas en la India en relación con las recomendaciones de la Organización Mundial de la Salud (OMS) y los esfuerzos nacionales de regulación para controlar las ventas de los antibióticos.

**Métodos:**

Se extrajeron datos sobre los volúmenes de ventas de antibióticos sistémicos en la India de una base de datos de ventas de empresas de investigación de mercado. Se comparó la cuota de mercado de las ventas de antibióticos en 2020 por categoría AWaRe (Access, Watch and Reserve) de la OMS y para los que estaban sometidos a controles regulatorios nacionales adicionales. También se analizaron las ventas de combinaciones en dosis fijas que estaban: formalmente aprobadas para su comercialización o tenían un certificado de no objeción; en la lista nacional de medicamentos esenciales; y en la lista de antibióticos no recomendados de la OMS.

**Resultados:**

En la India se comercializaron 78 antibióticos en dosis única y 112 antibióticos combinados en dosis fijas, que representaron 7,6 y 4,5 mil millones de unidades estándar de ventas totales, respectivamente. Los antibióticos de acceso, vigilancia y reserva supusieron 5,8, 5,6 y 0,1 mil millones de unidades estándar de las ventas totales del mercado, respectivamente. Todos los antibióticos controlados adicionalmente fueron antibióticos de vigilancia y reserva (23,6 %; 2,9 mil millones de unidades estándar de las ventas totales). Las combinaciones en dosis fijas incluidas en la lista no recomendada de la OMS se comercializaron en 229 formulaciones, de las que 114 (49,8 %) no tenían registro de aprobación formal ni certificado de no objeción. Mientras que no había combinaciones en dosis fijas no recomendadas en la lista nacional de medicamentos esenciales, 13 de las 20 combinaciones en dosis fijas de antibióticos más vendidas no estaban recomendadas por la OMS.

**Conclusión:**

Se debe investigar y controlar activamente la venta de medicamentos del grupo Watch y de antibióticos prohibidos o no aprobados en la India. Asimismo, se debe auditar la base de pruebas que sustenta las aprobaciones formales y los certificados de no objeción para las combinaciones en dosis fijas no recomendadas.

## ملخص

الغرض تحليل مبيعات تركيبة الجرعة الثابتة، والمضادات الحيوية المفردة في الهند فيما يتعلق بتوصيات منظمة الصحة العالمية (WHO)، والجهود التنظيمية الوطنية للتحكم في مبيعات المضادات الحيوية.

الطريقة قمنا باستخراج بيانات عن أحجام مبيعات المضادات الحيوية الجهازية في الهند من قاعدة بيانات مبيعات شركة أبحاث السوق. قمنا بمقارنة الحصة السوقية لمبيعات المضادات الحيوية في عام 2020 حسب فئة AWaRe (الوصول والمراقبة والاحتياطي) التابعة لمنظمة الصحة العالمية (WHO)، وتلك التي تخضع للضوابط التنظيمية الوطنية الإضافية. كما قمنا أيضًا بتحليل مبيعات تركيبات الجرعة الثابتة التي تم: الموافقة عليها رسميًا للتسويق، أو لديها شهادة عدم ممانعة؛ على قائمة الأدوية الأساسية الوطنية؛ وعلى قائمة منظمة الصحة العالمية (WHO) للمضادات الحيوية غير الموصى بها.

النتائج تم تسويق مضادات حيوية منها 112 تركيبة ذات جرعة ثابتة، و78 مضادًا حيويًا ذات تركيبة منفردة في الهند، وهو ما يمثل 7.6 و4.5 مليار وحدة قياسية من إجمالي المبيعات على الترتيب. تضم المضادات الحيوية Access وWatch وReserve 5.8 و5.6 و0.1 مليار وحدة قياسية من إجمالي مبيعات السوق على الترتيب. جميع المضادات الحيوية الإضافية الخاضعة للرقابة كانت Watch وReserve (%23.6؛ 2.9 مليار وحدة قياسية من إجمالي المبيعات). تركيبات الجرعة الثابتة الواردة في القائمة غير الموصى بها لدى منظمة الصحة العالمية (WHO) تم تسويقها في 229 مستحضرًا، مع 114 مستحضرًا (49.8%) ليس لها سجل بالموافقة الرسمية أو شهادة عدم ممانعة. على الرغم من عدم وجود تركيبات جرعة ثابتة غير موصى بها في القائمة الوطنية للأدوية الأساسية، فإن 13 من الـ 20 تركيبة جرعة ثابتة من المضادات الحيوية الأكثر مبيعًا لم تكن موصى بها من جانب منظمة الصحة العالمية (WHO).

الاستنتاج إن بيع أدوية مجموعة Watch، والمضادات الحيوية المحظورة أو التي لم تحصل على الموافقة، يحتاج إلى تحقيق وإنفاذ فعال في الهند. يجب مراجعة قاعدة الأدلة التي تقوم عليها الموافقات الرسمية، وشهادات عدم الممانعة لتركيبات الجرعة الثابتة غير الموصى بها.

## 摘要

**目的:**

根据世卫组织 (WHO) 推荐的药物目录和控制抗生素销售的国家监管措施,分析印度固定剂量组合抗生素和单一抗生素的销量。

**方法:**

我们从一家市场调研公司的销售数据库中提取了印度全身性抗生素的销量数据。我们比较了 2020 年按 WHO AWaRe 【可广泛使用 (Access)、谨慎使用 (Watch) 和保留使用 (Reserve)】分级目录和其他国家监管规定分类的抗生素的销售量市场份额。我们还分析了固定剂量组合抗生素(正式批准上市或者具有无异议证明;全国基本药物目录;世卫组织不推荐的抗生素目录)的销量。

**结果:**

印度有 78 种单一抗生素和 112 种固定剂量组合抗生素上市, 总销量分别为 76 和 45 亿标准单位。可广泛使用 (Access)、谨慎使用 (Watch) 和保留使用 (Reserve) 抗生素的市场总销量分别为 58、56 和 1 亿标准单位。所有额外受监管抗生素均为谨慎使用 (Watch) 和保留使用 (Reserve) 抗生素(占 23.6%,总销售额为 29 亿标准单位)。有 229 种非世卫组织推荐药物目录中的固定剂量组合抗生素上市,其中 114 种(49.8%)没有正式批准或无异议证明。虽然没有国家基本药物清单中不推荐的固定剂量组合抗生素,但在 20 种最畅销的固定剂量组合抗生素中,有 13 种是世卫组织不推荐使用的。

**结论:**

在印度还需对谨慎使用 (Watch) 类别的药物以及禁用或未批准的抗生素的销量进行积极调查并采取措施。应对不推荐使用的固定剂量组合抗生素取得的正式批准和无异议证明进行审查。

## Резюме

**Цель:**

Проанализировать продажи комбинированных препаратов с фиксированными дозами и отдельных антибиотиков в Индии в соответствии с рекомендациями Всемирной организации здравоохранения (ВОЗ) и мерами национального регулирования по контролю за продажами антибиотиков.

**Методы:**

Авторы извлекли данные об объемах продаж системных антибиотиков в Индии из базы данных по продажам исследовательской компании. Они сравнили рыночную долю продаж антибиотиков в 2020 г. по категориям AWaRe (Access (Доступ), Watch (Наблюдение), Reserve (Резерв)) ВОЗ, а также антибиотиков, на которые распространяются дополнительные национальные регулирующие меры контроля. Авторы также проанализировали продажи комбинированных препаратов с фиксированными дозами, которые были официально одобрены для продажи или имели сертификат об отсутствии возражений, находились в национальном перечне основных лекарственных средств и в списке ВОЗ не рекомендованных для использования антибиотиков.

**Результаты:**

В Индии на рынке представлены 78 отдельных и 112 комбинированных препаратов с фиксированными дозами антибиотиков, что составило 7,6 и 4,5 млрд условных единиц от общего объема продаж соответственно. Антибиотики групп Access, Watch и Reserve составили 5,8; 5,6 и 0,1 млрд условных единиц от общего объема продаж на рынке соответственно. Все дополнительно контролируемые антибиотики относились к антибиотикам групп Watch и Reserve (23,6%; 2,9 млрд условных единиц от общего объема продаж). Комбинированные препараты с фиксированными дозами, включенные в список ВОЗ не рекомендованных для использования антибиотиков, продавались в виде 229 лекарственных форм, при этом 114 форм (49,8%) не имели официального одобрения или сертификата об отсутствии возражений. Хотя в национальном перечне основных лекарственных средств не было нерекомендованных комбинированных препаратов с фиксированными дозами, 13 из 20 наиболее продаваемых комбинированных антибиотиков с фиксированными дозами не были рекомендованы ВОЗ.

**Вывод:**

Продажа антибиотиков группы Watch, а также продажа запрещенных или не имеющих одобрения антибиотиков требует активного расследования и правоприменения в Индии. Необходимо проверить доказательную базу, лежащую в основе официальных одобрений и сертификатов об отсутствии возражений для нерекомендованных комбинированных препаратов с фиксированными дозами.

## Introduction

The overuse and misuse of antibiotics contribute to the development of antimicrobial resistance, eroding the drugs’ effectiveness.^1^ Consequently, efforts continue to be made to improve antibiotic stewardship internationally and at country and local levels by organizations including the World Health Organization (WHO). WHO produced its first AWaRe classification in 2017,^2^ categorizing individual antibiotics as Access (to be widely available and affordable), Watch (to be used only for specific indications because their use increases the potential for emergence of antimicrobial resistance), and Reserve (for situations when all other alternatives have failed).

India has also taken several steps to tackle antimicrobial resistance.^3^^–^^6^ Initiatives include tightening restrictions on the sales of some prescription-only medicines, listed in and covered by Schedule H1 of the Drugs and Cosmetics Rules 1945. The restrictions, which were inserted into regulations in 2013, cover 33 single antibiotics and, with some exceptions, cover preparations which contain those antibiotics.^7^ Nevertheless, one of the highest rates of antibiotic resistance worldwide has been reported for India.^8^ The infectious disease mortality rate in India is 216.4 per 100 000 persons (authors’ calculation based on Global Burden of Disease 2019 data for India),^9^ with an estimated 56 524 neonatal deaths annually attributable to sepsis due to antibiotic resistance.^10^ India has the second highest overall antibiotic consumption worldwide, with a 47% increase between 2010 and 2020 from 5 411 to 7 976 million defined daily doses.^11^ Greater consumption has been driven by over-the-counter availability of cheap antibiotics in the context of weak compliance and enforcement of prescription-only regulations.^12^

In revising the AWaRe classification in 2019, WHO added a list of not-recommended fixed-dose combination antibiotics noting that their use “is not evidence-based, nor recommended in high-quality international guidelines. WHO does not recommend their use in clinical practice.”^2^ Fixed-dose combination products comprise two or more drugs combined in a fixed ratio of doses and available in a single dosage form. Fixed-dose combinations containing more than one antibiotic are effective in some well-studied situations, for example tuberculosis treatment. Fixed-dose combinations containing an antibiotic plus other non-antibiotic drugs also have proven effectiveness: for example, amoxycillin and clavulanic acid, a formulation that prevents bacteria degrading the amoxycillin. However, many antibiotic fixed-dose combinations are of unknown effectiveness.^13^

India is the country with the highest number of fixed-dose combination antibiotics marketed in the world.^14^ The central government has responded to long-standing official concerns about their proliferation^15^ and weak regulation,^16^ with several measures to control unapproved fixed-dose combinations (Box 1). These measures include the prohibition of the manufacture, distribution and sale of some fixed-dose combinations before WHO published its not-recommended list.

Box 1 **Summary of measures to control unapproved fixed-dose combinations in India, 2020**

India has a federal system of government, with drug regulatory functions divided between central and regional authorities. Regional authorities (states and union territories) grant licences for manufacturing, selling and distributing drugs. Before manufacturing licences for new drugs can be granted, including those that meet the definition of a fixed-dose combination, manufacturers must have obtained prior approval from the central regulatory authority, the Central Drugs Standard Control Organization headed by the Drugs Controller General (India), for a period of 4 years.

In March 2016^17^ (and June 2017)^18^ the government issued official notifications banning many fixed-dose combinations which had been licensed for manufacture without prior central approval, following the recommendations of an ad hoc technical assessment committee set up by the Ministry of Health and Family Welfare (Kokate committee).^19^^–^^21^ The March 2016 bans were immediately suspended after a successful challenge by representatives of the pharmaceutical industry in the Delhi High Court. On appeal, the banned drugs were remitted by the Supreme Court in December 2017 for reassessment by the Drugs Technical Advisory Board,^22^ the statutory adviser to central and state governments on technical matters arising out of the Indian 1940 Drugs and Cosmetics Act. The fixed-dose combinations were then reassessed by a Drugs Technical Advisory Board subcommittee^23^ and fresh bans followed in September 2018.^24^ Yet more fixed-dose combinations were banned in January 2019^25^ following the recommendations of another Drugs Technical Advisory Board subcommittee which had reported publicly in 2015.^26^

Alongside bans, the central regulatory authority has since 2015 also issued no-objection certificates for many antibiotic and other fixed-dose combinations which had been evaluated as rational by the Kokate committee.^27^^,^^28^ It appears that manufacturers with state licences are entitled to apply for no-objection certificates which, when issued, would amount to de facto or effective approval of the formulations, despite the absence of a formal central approval.

The primary aim of this study was to examine how sales of antibiotics in India align with WHO recommendations and the Indian government’s regulatory efforts. Given the importance of essential medicines to satisfy priority population needs, a secondary aim was to ascertain the market share of those antibiotic fixed-dose combinations that are included in India’s national list of essential medicines published in 2015.^29^

## Methods

### Data sources

This descriptive study was based on market sales data for 2020 and official documents. We obtained sales data from a data set compiled by the pharmaceutical market research company Pharmasofttech, known as PharmaTrac.^30^ PharmaTrac provides monthly pack-wise values and volumes, drug names, dosage forms and strengths of medicine in the Indian pharmaceutical market. The data are collected from a sample of 10 000 stockists capturing their sales across 30 regions of the country and extrapolated to reflect overall sales in the private market. Costs of medicines in India are primarily covered by out-of-pocket payments although the situation has been improving since 2013. According to the national health account estimates for India, private expenditure on medicines at retail pharmacies was around 1503.3 billion Indian rupees (INR), 90% of the total expenditure on medicines of INR 1666.3 billion in 2013–2014^31^ and had fallen to around 69% (INR 1164.0/INR 1676.5 billion) by 2017–2018.^32^ Data on public medicine procurement were not publicly available at the national level.

Official documents comprised the 2019 WHO list of AWaRe group antibiotics and not-recommended fixed-dose combinations;^2^ the additional Indian regulations on the sales of some prescription-only medicines published in 2013 (Schedule H1);^7^ and documents published by the Indian authorities. These documents specify fixed-dose combinations of: (i) drugs approved for marketing;^33^ (ii) drugs given a no-objection certificate;^27^^,^^28^ (iii) drugs prohibited from manufacture;^24^^,^^25^^,^^34^ and (iv) drugs in India’s national list of essential medicines published in 2015.^29^

In the official documents, we identified and extracted information on systemic antibiotics. We excluded antimicrobial fixed-dose combinations that did not include any antibiotic, such as antivirals, antifungals and fixed-dose combinations indicated for tuberculosis where there is a well-established evidence base for use. We excluded topical preparations, kits and combi-kits (packaging including two or more medicines to be used concomitantly). We included fixed-dose combinations comprising at least one antibiotic; combinations of two or more antibiotics; and combinations of an antibiotic with another antimicrobial (dual antimicrobials). 

Raw sales data for the study can be accessed through AIOCD Pharmasofttech AWACS Pvt. Ltd, a joint venture of All Indian Origin Chemists & Distributors Ltd (AIOCD Ltd) and Trikaal Mediinfotech Pvt. Ltd.^35^

### Data analysis

We converted volumes of sales of antibiotics ‒ which are reported as number of packs ‒ to standard units, where one standard unit was one tablet or capsule, one injection vial or one bottle of oral medicine. For example, if 100 packs of 10 tablets each of a medicine were sold in the marketplace, we computed that 1000 standard units (100 × 10 tablets) had been sold.

We combined the sales volume data and the data extracted from the official documents with WHO recommendations. We assigned the AWaRe category of fixed-dose combinations according to the antibiotic in the highest AWaRe category; for example, combinations of an Access antibiotic with a Watch antibiotic were assigned to the Watch category. When sales volume levels are low, PharmaTrac groups products according to an antibiotic, an antibiotic class or a therapeutic group. Antibiotic classes that span more than one AWaRe category (such as cephalosporins) or therapeutic groups (such as antidiarrhoeal drugs) could not be assigned an AWaRe category; we classified these drugs as Unclassified.

The listings of drugs approved by the central regulatory authority, drugs with a no-objection certificate and drugs on the national list of essential medicines are for formulations of fixed-dose combinations with specific dosage forms and strengths. If a tablet was specified as approved, then we coded all types of tablets as approved. If a form of tablet was specified (such as sustained release), then we coded only the specified form of tablet as approved, while other types (such as film-coated tablets) were coded as not approved. We followed the same approach for strengths; for example, if only the strength 500 mg and 125 mg was approved, we only coded this strength as approved and other strengths as not approved. For the purpose of the analysis, we grouped identified formulations of fixed-dose combinations according to active ingredients, irrespective of salt form. Lactic acid, lactobacillus, lactic acid bacillus and *Lactobacillus acidophilus* were considered equivalent. We searched the sources for alternative spellings (such as amoxicillin, amoxycillin; or sulbactam, sulbactum, salbactum). For some drugs, the PharmaTrac data set did not provide information on strength, or grouped products according to an antibiotic class or therapeutic group. In these cases we could not determine the national regulatory authority approval and national list of essential medicines listing; we therefore defined these drugs as undetermined.

We calculated the number of antibiotic drugs sold, the volume sold and the percentage market share for all antibiotic fixed-dose combinations and single drugs in the Indian marketplace by Access, Watch and Reserve groups and by additionally controlled listing (Schedule H1). We then analysed sales volume data for fixed-dose combinations that were (i) on the WHO not-recommended list; (ii) formally approved by the national regulatory authority or de facto approved through a no-objection certificate; (iii) banned; and (iv) on the national list of essential medicines 2015.

## Results

In 2020, 12.1 billion standard units of systemic antibiotics were sold in India. There were 78 single antibiotics and 112 fixed-dose combination antibiotics on the market, accounting for 62.7% (7.6 billion standard units) and 37.3% (4.5 billion standard units) of total antibiotic sales (12.1 billion standard units), respectively. Dual antimicrobials accounted for 61.6% (69/112) of fixed-dose combinations in 2020. Full data for all the results are in the authors’ data respository.^36^

### WHO AWaRe groups 

Based on the 2019 WHO AWaRe classification,^2^ we determined that Access, Watch and Reserve antibiotics accounted for 47.9% (5.8 billion standard units), 46.7% (5.6 billion standard units) and 1.0% (0.1 billion standard units) of sales, respectively.

Of the 78 single antibiotics sold, 50 were Watch group and nine were Reserve. The market share of Watch single antibiotics was 34.1% (4.1 billion standard units of all antibiotic sales), exceeding that of the Access group drugs (27.7%; 3.3 billion standard units). The market share of Reserve single antibiotics was 1.0% (0.1 billion standard units) (Table 1). 

**Table 1:** Sales volume and market share of Access, Watch and Reserve single drugs and fixed-dose combinations of systemic antibiotics, India, 2020

Formulations	WHO AWaRe group^a^													
	All			Access			Watch			Reserve			Unclassified^b^	
	Total no. of drugs marketed	Total sales volume, billions of standard units		No. of drugs marketed	Sales volume, billions of standard units (% of all)		No. of drugs marketed	Sales volume, billions of standard units (% of all)		No. of drugs marketed	Sales volume, billions of standard units (% of all)		No. of drugs marketed	Sales volume, billions of standard units (% of all)
Single drugs	78	7.6		21	3.3 (27.7)		50^c^	4.1 (34.1)		9^c^	0.1 (1.0)		0	0 (NA)
Fixed-dose combinations	112	4.5		39	2.4 (20.2)		70	1.5 (12.7)		3	< 0.1(< 0.1)		NA	0.5 (4.4)
Total	NA	12.1		NA	5.8 (47.9)		NA	5.6 (46.7)^d^		NA	0.12 (1.0)		NA	0.5 (4.4)

AWaRe: Access, Watch and Reserve; NA: not applicable; WHO: World Health Organization.

^a^ Based on WHO AWaRe antibiotic stewardship classification, 2019.^2^

^b^ Unclassified antibiotics included data grouped by PharmaTrac as antibiotic classes and therapeutic groups, where it was not possible to assign an AWaRe category.

^c^ Two antibiotics were counted under both Watch and Reserve groups because their AWaRe classification is dosage-form specific: fosfomycin oral (Watch), fosfomycin parenteral (Reserve); minocycline oral (Watch), minocycline parenteral (Reserve).

^d^ Watch antibiotics accounted for 46.72% (34.05% single drugs + 12.67% fixed-dose combinations).

Data source: Sales volumes were from the Pharmasofttech (PharmaTrac) database, 2020.^30^

Among the 112 fixed-dose combination antibiotics marketed, 70 were Watch group and three were Reserve. The market share of Access group combinations (20.2%; 2.4 billion standard units) exceeded the Watch group combinations (12.7%; 1.5 billion standard units). The market share of Reserve group combinations was small, with only three combinations marketed in this category: cefixime and linezolid (Watch and Reserve); cefuroxime and linezolid (Watch and Reserve); and ceftazidime and avibactam (Reserve).

We found that 21 single antibiotics marketed were on the additionally controlled list (Schedule H1), accounting for 17.3% (2.1 billion standard units) of antibiotic sales. Apart from one drug classified as Reserve (faropenem), all were classified as Watch.^36^ A total of 38 Watch and Reserve single antibiotics marketed in India were not included in or covered by the additionally controlled drug list. 

There were 41 combinations of 13 additionally controlled antibiotics marketed, accounting for 6.4% (0.8 billion standard units) of the antibiotic market. Apart from two Reserve fixed-dose combinations (cefixime and linezolid; ceftazidime and avibactam), all were classified as Watch.^36^ However, 34 Watch and Reserve fixed-dose combinations marketed were not covered by the additionally controlled drug listing.

### WHO not-recommended status

Of the 103 not-recommended fixed-dose combination antibiotics listed by WHO, 57 were marketed in India in 2020, comprising 41.5% of total antibiotic fixed-dose combination sales (1.9/4.5 billion standard units).^36^ Thirteen of the top-20 selling fixed-dose combination antibiotics were on the WHO not-recommended list (Table 2). These drugs comprised 37.9% of total antibiotic fixed-dose combination sales (1.7/4.5 billion standard units) and were mostly dual antimicrobials (8/13 formulations) and Watch antibiotics (10/13 formulations). Cefpodoxime and ofloxacin was the only combination for which all marketed formulations had a record of formal regulatory authority approval (1/1 formulation). The remaining 19 fixed-dose combinations were available in numerous unapproved formulations.

**Table 2:** Profile of top-20 selling fixed-dose combinations of systemic antibiotics, India, 2020

Fixed-dose combinations	Sales volume, millions of standard units (% of all)	Dual antimicrobial	WHO AWaRe group^a^	WHO not-recommended list^b^	No. of formulations marketed		
					Total	Approved^c^	No-objection certificate^d^
Amoxycillin and clavulanic acid	1225.9 (27.3)	No	Aware	No	42	19	0
Ampicillin and cloxacillin	431.4 (9.6)	Yes	Aware	Yes	10	0	3
Ofloxacin and ornidazole	314.2 (7.0)	Yes	Watch	Yes	29	1	0
Cefixime and ofloxacin	278.4 (6.2)	Yes	Watch	Yes	11	6	0
Doxycycline and lactobacillus	227.0 (5.0)	No	Aware	No	3	2	No
Trimethoprim and sulfamethoxazole	214.7 (4.8)	Yes	Aware	No	5	1	0
Cefpodoxime and clavulanic acid	161.9 (3.6)	No	Watch	Yes	17	7	No
Amoxycillin and cloxacillin	88.4 (2.0)	Yes	Aware	Yes	8	0	No
Ampicillin and dicloxacillin	82.2 (1.8)	Yes	Aware	Yes	3	0	2
Ciprofloxacin and tinidazole	77.8 (1.7)	Yes	Watch	Yes	4	0	2
Norfloxacin and tinidazole	71.7 (1.6)	Yes	Watch	No	3	1	1
Cefixime and clavulanic acid	61.5 (1.4)	No	Watch	Yes	12	2	No
Cefpodoxime and ofloxacin	49.4 (1.1)	Yes	Watch	Yes	1	1	0
Ceftriaxone and sulbactam	47.9 (1.1)	No	Watch	Yes	6	5	No
Cefuroxime and clavulanic acid	47.4 (1.1)	No	Watch	Yes	9	5	No
Ceftriaxone and tazobactam	37.7 (0.8)	No	Watch	Yes	6	5	No
Ofloxacin and metronidazole	30.0 (0.7)	Yes	Watch	No	10	0	No
Piperacillin and tazobactam	27.4 (0.6)	No	Watch	No	3	3	1
Cefixime and dicloxacillin and lactobacillus	25.8 (0.6)	Yes	Watch	Yes^e^	7	0	No
Ofloxacin and flavoxate	21.1 (0.5)	No	Watch	No	1	0	No

AWaRe: Access, Watch and Reserve; WHO: World Health Organization.

^a^ Based on WHO AWaRe antibiotic stewardship classification: Access (to be widely available and affordable), Watch (to be used only for specific indications because their use increases the potential for emergence of antimicrobial resistance) and Reserve (for the situations when all other alternatives have failed).^2^

^b^ Based on WHO list of fixed-dose combination antibiotics not recommended for use in clinical practice.^2^

^c^ Based on lists of drugs with Indian Central Drugs Standard Control Organization approval.^33^^,^^37^

^d^ Based on lists of drugs with no-objection certificates.^27^^,^^28^ “No” signifies that no formulations of the particular fixed-dose combination were given a no-objection certificate, while “0” signifies that some formulations of the fixed-dose combination were given a no-objection certificate but none of them were marketed.

^e^ Cefixime and dicloxacillin is WHO not-recommended. We considered cefixime and dicloxacillin and lactobacillus to be also not-recommended although this combination itself is not listed as WHO not-recommended.

Notes: Total sales volume was 4497.3 million standard units. Sales volumes were from the Pharmasofttech (PharmaTrac) database, 2020.^30^

**Fig. 1:**
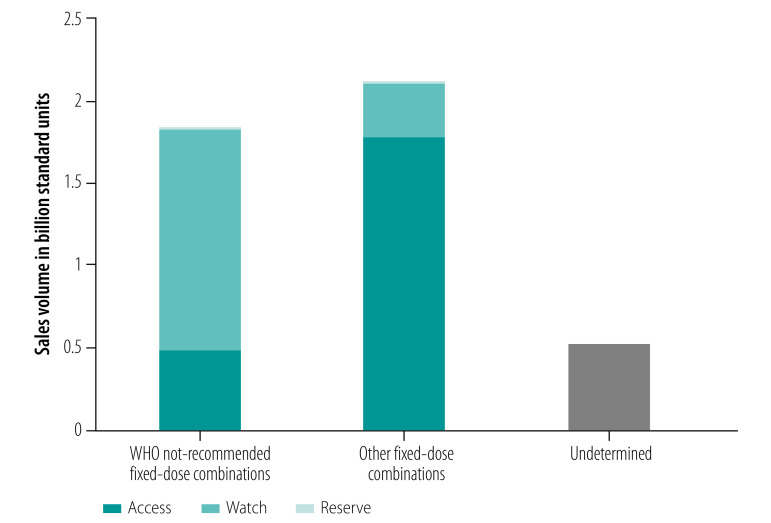
Sales volume of fixed-dose combination antibiotics, by WHO AWaRe groups and not-recommended status, India, 2020

AWaRe: Access, Watch and Reserve; WHO: World Health Organization.

Note: Sales volumes were from the Pharmasofttech (PharmaTrac) database, 2020.^30^ The AWaRe antibiotic stewardship classifies drugs as: Access (to be widely available and affordable), Watch (to be used only for specific indications because their use increases the potential for emergence of antimicrobial resistance) and Reserve (for the situations when all other alternatives have failed). Not-recommended drugs are fixed-dose combination antibiotics not recommended for use in clinical practice.^2^ Drugs included in the undetermined group are drugs grouped by PharmaTrac as antibiotic classes and therapeutic groups, where it was not always possible to determine WHO not-recommended status.

Overall, WHO not-recommended fixed-dose combinations were a much higher proportion of Watch antibiotics than fixed-dose combinations not included on the WHO list (Fig. 1). Moreover, two of the three marketed Reserve fixed-dose combinations were WHO not-recommended (cefixime and linezolid; cefuroxime and linezolid).

### Indian regulatory status

The WHO not-recommended list and government banning orders apply to combinations, whereas Indian regulatory authority formal approvals and no-objection certificates are in reference to specific formulations. More than half of the formulations marketed (58.0%; 229/395 formulations) were of WHO not-recommended combinations (Table 3). These drugs related to 57 combinations on the not-recommended list and accounted for 41.5% of antibiotic fixed-dose combination sales (1.9/4.5 billion standard units).^36^ Of the 229 not-recommended formulations: 63 (27.5%) formulations had a record of formal central approval; 15 (6.6%) formulations had been given a no-objection certificate; 37 (16.2%) formulations had been banned; and 114 (49.8%) formulations had no record of approval, no-objection certificate or ban.^36^ Of 166 formulations of other fixed-dose combinations, 32 (19.3%) had a record of formal central approval; 7 (4.2%) had been given a no-objection certificate; 8 (4.8%) had been banned; and 119 (71.7%) formulations had no record of approval, no-objection certificate or ban.^36^

**Table 3:** Sales volume and market share of fixed-dose combination antibiotics, by not-recommended and regulatory status, India, 2020

Formulations	All fixed-dose combinations			WHO not-recommended fixed-dose combinations^a^			Other fixed-dose combinations	
	No. of formulations marketed	Sales volume, billion standard units (% of all)		No. of formulations marketed	Sales volume, billion standard units (% of all)		No. of formulations marketed	Sales volume, billion standard units (% of all)
**Total**	> 395	4.5 (100.0)		> 229	1.9 (41.5)		> 166	2.6 (58.5)
**Known formulations **	395	3.7 (81.5)		229	1.8 (40.7)		166	1.8 (40.8)
Approved^b^	117	3.0 (65.9)		78	1.5 (32.2)		39	1.5 (33.7)
Banned	42	< 0.1 (0.2)		37	< 0.1 (0.2)		5	< 0.1 (0.1)
Not approved or banned	236	0.7 (15.4)		114	0.4 (8.3)		122	0.3 (7.1)
**Undetermined^c^**	NA	0.8 (18.5)		NA	< 0.1 (0.7)		NA	0.8 (17.7)

NA: Not applicable; WHO: World Health Organization.

^a^ Based on WHO list of fixed-dose combination antibiotics not recommended for use in clinical practice.^2^

^b^ Drugs with Indian Central Drugs Standard Control Organization approval plus no-objection certificate.

^c^ Undetermined drugs include data grouped by PharmaTrac as antibiotic classes and therapeutic groups, where it was not always possible to determine to which group to assign the data.

Data source: Sales volumes were from the Pharmasofttech (PharmaTrac) database, 2020.^30^

In the antibiotic fixed-dose combination market, approved formulations (formal central approval plus no-objection certificate) accounted for 65.9% of total sales volume (3.0/4.5 billion standard units): 32.2% (1.5 billion standard units) for not-recommended fixed-dose combinations and 33.7% (1.5 billion standard units) for other fixed-dose combinations. Banned formulations accounted for 0.2% of sales volume (< 0.1/4.5 billion standard units). However, 15.4% of sales (0.7/4.5 billion standard units) were of formulations without a record of approval (or ban). For the remaining 18.5% (0.8/4.5 billion standard units), it was not possible to determine whether approvals had been obtained.

The 2015 Indian national list of essential medicines does not include any formulations of WHO not-recommended combinations. A total of 17 formulations of fixed-dose combinations (12 Access and five Watch formulations) listed as essential in India were marketed, with a market share of 26.7% (1.2/4.5 billion standard units).

## Discussion

Private market sales of systemic antibiotics in India were vast. We found that combinations not recommended by WHO accounted for 41.5% of sales of fixed-dose combinations and were predominately Watch group combinations. Another major finding is that 236 out of 395 formulations of fixed-dose combinations were marketed without formal central approval, accounting for 15.4% of fixed-dose combination sales. Mirroring previous literature,^38^ we found higher sales of Watch single antibiotics compared with Access single antibiotics, while the overall sales of Access antibiotics slightly exceeded sales of Watch antibiotics. This finding differs from an analysis of 2015 data, based on a different data set and method, which showed overall sales being dominated by Watch antibiotics.^39^

Before WHO introduced its AWaRe classification, India had already taken steps to regulate sales of antibiotics including single and fixed-dose combination antibiotics. In addition to prescription-only status, these regulations require a package warning and mandate pharmacists to keep records of the prescriber, patient and drug dispensed (Schedule H1). Previous research found this intervention was effective in reducing sales.^40^ However, the regulations do not include all antibiotics categorized as Watch and Reserve by WHO. Single and fixed-dose combination antibiotics falling under the additionally controlled list accounted for 23.62% (17.26% single drugs + 6.36% fixed-dose combinations; 2.9 billion standard units) of antibiotic sales in 2020 compared with the 47.8% (5.8 billion standard units) market share attributed to Watch and Reserve antibiotics. Compared with Access antibiotics, Watch and Reserve agents have a higher risk of inducing resistance in bacterial pathogens.^2^ Adding all Watch and Reserve antibiotics to the regulations could therefore potentially reduce their use. However, while the AWaRe classification has been updated biennially since 2017, changes to the Indian regulations follow a legislative process and only two (non-antibiotic) drugs have been added since 2013.

To tackle the proliferation of fixed-dose combinations which have been given manufacturing licences by individual states in India before prior approval for marketing from the central regulator,^41^ the government banned many combinations, including some antibiotic fixed-dose combinations listed as not recommended by WHO. However, some WHO not-recommended combinations have either been previously approved by the central regulator or assessed as rational and given a no-objection certificate to allow their continued marketing. Information needs to be published on the evidence base which underpins approvals in India and the WHO not-recommended list of antibiotics. In India, many antibiotic fixed-dose combinations which are pharmacologically related to those on the not-recommended list are marketed.

Many fixed-dose combinations banned by the government continued to be marketed in India. For instance, two of the three Reserve fixed-dose combinations on the market are WHO not-recommended (cefixime and linezolid; cefuroxime and linezolid). Although neither of these drugs had a record of prior central approval, and both were banned in 2018, there was still evidence of sales in 2020. This finding aligns with a study in India examining 14 of 26 fixed-dose combinations banned in September 2018 which found that while sales decreased following the ban, some fixed-dose combinations remained on the market in significant volumes and sales of pharmacologically related, but not-banned, fixed-dose combinations increased.^42^

Although the recent initiatives in India led to banning orders and de facto approvals through no-objection certificates, many antibiotic combinations in the market remain unapproved.

Our detailed analysis of information on regulatory status of fixed-dose combinations in India collated from various official documents was restricted to publicly available documents. Limitations of the study arise from the quality of market sales data that include misreported or missing data on strengths of marketed formulations. We could not determine approval status for almost one fifth of sales volume in 2020 because of missing data (strengths of formulations) and due to sales being recorded according to antibiotic or therapeutic class rather than formulation. Further analysis of the undetermined category could identify antibiotics which may have been approved, although our sensitivity analysis of formulations with missing strengths shows these drugs accounted for less than 1% of market volume.

Several policy implications arise from the results. First, there is a need for greater transparency and review of antibiotic fixed-dose combinations marketed in India. Indian authorities should review the evidence for marketed formulations of combinations not recommended by WHO but which have a record of central approval or no-objection certificate.^36^ In view of the size of the Indian antibiotic market and country’s antibiotic resistance profile, the Indian authorities and WHO should consult to understand and potentially evolve each other’s position. The results of the review and consultation should be published. 

Second, the effectiveness and enforcement of the regulatory process in India needs to be strengthened. The reasons for the high sales of Watch antibiotics need to be investigated by the Indian authorities. The inclusion of all Watch and Reserve antibiotics in the additionally controlled drugs Schedule H1 should be considered. Unapproved and banned formulations must be removed from the market. In line with the views of a former joint secretary in the health ministry,^43^ criminal proceedings should be considered against the manufacturers of formulations marketed in 2020 without a record of prior central approval in circumstances where such approval was legally required but had not been granted. Proceedings should also be considered where banned antibiotic fixed-dose combinations remain on the market.

Third, given that antimicrobial resistance is a global concern, there is a need to intensify international collaboration on antibiotic fixed-dose combination assessments. Initiatives could include evaluations of safety and efficacy and of antimicrobial resistance potential, and to regulate manufacture and sales of fixed-dose combinations, as recommended in a review of the fixed-dose combination amoxycillin and cloxacillin.^13^ A study of antibiotic fixed-dose combination consumption in 75 countries reiterated these views, adding that the rationale for fixed-dose combination use also needed to be explored.^14^ Common methods for assessment of fixed-dose combinations, taking account of infection priorities and resistance profiles, should be developed collaboratively and made publicly available. These measures could draw on initiatives such as the European Union’s Medicines for All initiative^44^^,^^45^ and the United States Food and Drug Administration’s PEPFAR-linked review.^46^ WHO’s not-recommended list is an important guidance step, but the methods by which included fixed-dose combinations were selected and the evidence supporting the listing should be publicly available, similar to those of the AWaRe classification.^47^
